# Expression and clinical significance of multidrug resistance proteins in brain tumors

**DOI:** 10.1186/1756-9966-29-122

**Published:** 2010-09-05

**Authors:** Zhenhua Guo, Jin Zhu, Lihua Zhao, Qing Luo, Xianqing Jin

**Affiliations:** 1 Department of Oncology, Children's Hospital of Chongqing Medical University, Chongqing, China

## Abstract

**Background:**

To investigate the mechanisms of multidrug resistance of brain tumors, to identify the site of cellular expression of P-gp in human brains in situ and to morphologically determine whether an association may exist between P-gp and caveolin-1.

**Methods:**

Immunohistochemistry was used to detect the expression and location of P-glycoprotein (P-gp), Multidrug resistance-associated protein (MDR), Lung resistance-related protein (LRP), Topoisomerase II (Topo II) and Glutathione-S-π (GST-π) in 30 patient tumor tissues and 5 normal brain tissues. The sections were subjected to double labeling for P-gp (TRITC labeled) and caveolin-1 (FITC labeled). The location and characteristics of expression of the two proteins in the blood brain barrier(BBB) was observed using a laser scanning microscope.

**Results:**

High expression of P-gp was detected in vessel walls and the tissue surrounding the vessels. However, expression of P-gp was low in tumor cells. The expression of the other 4 multidrug resistance proteins was not observed in the vessel walls. Laser scanning microscopy showed P-gp and caveolin-1 co-expression: the two proteins co-localized either in the luminal endothelial compartment or at the border of the luminal/abluminal compartments.

**Conclusion:**

Chemotherapeutics drugs are interrupted in the end-feet of neuroepithelial cells of the BBB by P-gp, which weakens the chemotherapeutic effect. P-gp marks the BBB, and the transporter is localized in the luminal endothelial compartment where it co-localizes with caveolin-1.

## Introduction

Currently, primary malignant brain tumors and brain metastases are still difficult to treat with cytotoxic agents. Even though new chemotherapeutic schedules have improved results of cancer treatment in other parts of the body (e.g., small-cell lung cancer, breast cancer, various leukemias), the efficacy of these new schedules in brain tumors remains poor [1]. In addition to the blood brain barrier(BBB), resistance mechanisms at the tumor cell level may include the intrinsic chemo-insensitivity of brain tumors. The BBB is a major impediment to the entry of many therapeutic drugs into the brain, and over the last decade, it has become clear that multispecific, xenobiotic transporters play a significant role at the BBB [2]. The major determinants of drug permeability across the BBB have long been thought to be based solely on lipophilicity and molecular weight. Although many anticancer drugs are highly lipophilic and relatively small, the permeation level of those drugs across the BBB is unexpectedly low [3]. This can be partially explained by the expression of P-glycoprotein (P-gp) [4,5]. P-glycoprotein (P-gp) is a 170-kDa transmembrane glycoprotein that is encoded by the human multidrug-resistance gene MDR1 and is an important functional component of the BBB [6]. P-glycoprotein is an adenosine triphosphate (ATP)-dependent pump. When the drug enters the cells, ATP hydrolysis provides the energy for active drug transport, enabling the transporter to function against steep concentration gradients. The drug and ATP initially bind to the protein at their respective binding sites, where ATP hydrolyzes to ADP and yields energy for extrusion of the drug [7]. The intracellular drug concentration remains at a low level, leading to tumor cell resistance. There are two different views about the exact location of P-gp in the BBB. One view is that the P-gp is only at the luminal endothelial membrane. This selective one-front localization suggests that P-gp plays a barrier protective role by extruding cytotoxic substances and drugs from the endothelial cells back into the bloodstream [8]. Another view is that the site of expression of P-gp is also in perivascular astrocytes in the human brain [9,10]. Moreover, recently studies have shown that P-gp is localized to caveolae and co-immunoprecipitates with caveolin-1 [11], an integral protein of the caveolae frame, suggesting that the two proteins might physically interact.

The purpose of the present study was to examine the mechanisms of multidrug resistance of brain tumors and the localization of P-gp in pediatric brain tumors. This in situ study was carried out on tumor tissues by immunohistochemistry using a monoclonal antibody against P-gp. In addition, double immunolabeling was carried out with antibodies to P-gp and caveolin-1 by immunofluorescence laser scanning confocal microscopy to ascertain whether there is any association between these molecules in the microvessels of brain tumors.

## Materials and methods

### Materials

This study included 30 samples of pediatric brain tumor tissues, including 19 astrocytomas, 8 ependymomas, 3 medulloblastomas. The patients were 20 boys and 10 girls ranging between 6 months and 12 years (median 7.6 years) who were undergoing tumor resection without chemotherapy for high grade (III-IV) tumors (10 cases) and low grade (I-II) tumors (20 cases), according to the grade of Malignancy of Brain Tumor in WHO in 2000 [12]. Five brain tissue samples from autopsies (patients died due to cardiovascular disease) were used as controls.

### Immunohistochemistry

Paraffin sections were first rehydrated, and then rehydrated sections were incubated with a 1:200 dilution of rabbit anti-human primary antibody against P-gp (Santa Cruz Biotechnology, Santa Cruz, CA), LRP (ABCOM Information Systems Pvt. Ltd, USA), MRP (Maixin Bio, Fuzhou, China), GST-π (Maixin Bio, Fuzhou, China), Topo II (ABCOM Information Systems Pvt. Ltd, USA), S-100 (Santa Cruz Biotechnology, Santa Cruz, CA) or control IgG (1:1000) overnight at 4°C. The tissue sections were washed in PBS and then incubated with a 1:100 dilution of biotinylated secondary sheep anti-rabbit or goat anti-rabbit IgG (Jingmei BioTech, Shenzhen, China). After washing with PBS, tissue sections were incubated with an avidin-biotin complex and developed in 0.075% (w:v) 3,3 diaminobenzidine (DAB). After lightly counterstaining with haematoxylin, the sections were dehydrated. P-gp, MRP, LRP, GST-π are expressed in the cell membrane and or cytoplasm, and Topo-II is expressed in the nucleus. A positive reaction is colored brown. The intensity of immunostaining around the stent struts was scored as follows: 0, no staining; 1, minor staining only; 2, moderate staining; and 3, heavy staining. Intensities of 2 and 3 were considered strongly positive and indicate that drug resistance would be induced by the resistance protein. Scoring was performed on every third strut in each vessel beginning with the strut closest to the top of the slide by an investigator blinded to the treatment allocation.

### Immune co-labeling of P-gp/caveolin-1

Tumor sections were first permeabilized in 0.01 M PBS containing 0.1% Triton X-100 (PBST) for 3 × 5 min followed by blocking in 10% goat serum in 0.01 M PBST. Antibodies were diluted in 3% bovine serum albumin in 0.01 M PBST. The anti-caveolin-1 antibody was used at concentrations between 0.2-5 mg/ml, and the anti-P-gp antibody was used at a concentration of 5-10 mg/ml. Prior to application to the tissue, the primary antibody was pre-incubated with the blocking peptide at 37°C for 3 h (caveolin-1, Santa Cruz Biotechnology, Santa Cruz, CA) or overnight at 4°C (P-gp). After thorough washing steps using PBS, sections were incubated for 1 h at room temperature with 5 mg/ml of each secondary antibody. Following washing with PBS, the sections were put on coverslips.

The sections were viewed under a Leica TCS-SP2 confocal laser scanning microscope (Leica Instruments Co., Ltd. German) using ×40 and ×63 oil-immersion objective lenses with either ×1 or ×2 zoom factors. On the double-immunolabeled sections, a sequential scan procedure was applied during image acquisition of the two fluorophores, Alexa Fluor 488 (excitation at 488 nm and detection range 500-535 nm; green fluorescence) and Alexa Fluor 568 (excitation at 568 nm and detection range 580-620 nm; red fluorescence). Confocal images were taken at 0.5-μm intervals through the z-axis of the section, and a total depth of 15-20 μm was covered. Images from individual optical sections and multiple serial optical sections were analyzed, recorded digitally and stored as TIFF files in Adobe Photoshop software.

All the investigation procedures on human tissues were carried out in accordance with the local institutional ethical committee policies. This study were approved by the relevant regulatory committee of the Chongqing Medical University.

### Statistical analysis

Statistical data were analyzed using the SPSS 10.03 software (SPSS, Chicago, IL, USA). The rank sum test was used to examine the differential expression of the 5 multidrug resistance proteins in brain tumors. Fisher definite probability methods were used to examine the differences between high grade and low grade tumors. Results were considered statistically significant at *P *< 0.05.

## Results

### Expression of 5 multidrug resistance proteins in brain tumors

In our study, we find the expression of multidrug resistance proteins in three different positions, including tumor cells, capillary vessels and interstitial cells.

In tumor cells, the strongly positive rate of P-gp, Topo II, GST-π, LRP and MRP were 26.67%, 23.33%,16.67%,3.33% and 3.33%, respectively (Tab 1). This difference was statistically significant (Rank sum test, P < 0.01), which shows that the positive expression of the 5 multidrug resistance proteins in brain tumors is not identical, and the positive expression of P-gp was observed to be the highest.

**Table 1:** Expression of the 5 multidrug resistance proteins in the tumor cells

Multidrug resistance protein	n	-	+	++	+++	Strongly positive rate (%)
P-gp	30	4	18	8	0	26.67
Topo II	30	13	10	7	0	23.33
GST-π	30	10	15	5	0	16.67
MRP	30	28	1	1	0	3.33
LRP	30	26	3	1	0	3.33

In tumor cells, the strongly positive rate of P-gp, Topo II, GST-π, LRP and MRP were 26.67%,23.33%,16.67%,3.33% and 3.33%, respectively. This difference was statistically significant (Rank sum test, P < 0.05)

**Figure 1:**
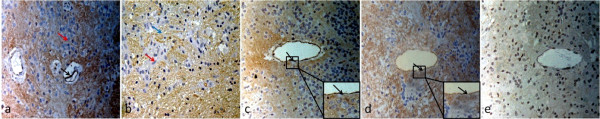
**The expression of P-gp and S-100 in brain tumors (astrocytoma),(×400)**. (a, b, c) The expression of P-gp is weak in tumor cells (red arrow), but strongly positive in capillary vessels (black arrow). (c) The expression of P-gp in the interstitial cells was related to the distance from the capillary wall. The expression of P-gp was stronger the nearer the cell was to the capillary wall (green arrow). (d) The expression of S-100 in brain tumors. Our study shows the expression of P-gp and S-100 are co- localized in the capillary endothelial cells and interstitial cells of tumor tissues. These findings suggest P-gp expression at the microvasculature is found at both the endothelium as well at the astrocyte end-feet at the microvasculature.

However, in our study, the expression of P-gp is weak in tumor cells but strongly positive in capillary vessels (Fig 1a, b and Fig 1c). Low positive expression of LRP, MRP, GST-π and Topo II was observed in capillary vessels (Tab 2). In the normal brain tissues, the expression of P-gp was strongly positive in the tissues surrounding the cerebral vessels, but no positive expression was observed in capillary vessels. The BBB contains capillary endothelial cells, basement membrane and the end-feet of astrocytes. The accurate structure is difficult to distinguish using ordinary light microscopy. In order to confirm the expression of P-gp in the end-feet of astrocytes, the S-100 protein was used to locate the end-feet of astrocytes by immunohistochemistry. The expression of the S-100 protein was positive in the capillary walls (Fig 1d). These findings suggest P-gp expression in the microvasculature is found at both the endothelium as well at the astrocyte end-feet at the microvasculature. In addition, the same results were observed in the interstitial cells.

**Table 2:** Expression of the 5 multidrug resistance proteins in the capillary walls of tumor tissues

Multidrug resistance protein	n	-	+	++	+++	Strongly positive rate (%)
P-gp	30	3	6	12	9	70.00
Topo II	30	23	5	2	0	6.67
GST-π	30	26	3	1	0	3.33
MRP	30	27	2	1	0	3.33
LRP	30	27	3	0	0	0.00

The expression of P-gp is strongly positive in capillary vessels. Low positive expression of LRP, MRP, GST-π and Topo II was observed in capillary vessels. This difference was statistically significant (Rank sum test, P < 0.01)

Otherwise, we find the expression of resistance proteins in interstitial cells are similar to the tumor cells. The positive expression of P-gp is highest(Tab 3), which suggests that P-gp is the main protein in tumor drug resistance. The expression of P-gp in the interstitial cells was related to the distance of the cells from the capillary wall. The nearer the cell was to the capillary wall, the stronger the expression of P-gp (Fig 1c).

**Table 3:** Expression of the 5 multidrug resistance proteins in the interstitial cells

Multidrug resistance protein	n	-	+	++	+++	Strongly positive rate(%)
P-gp	30	3	13	10	4	46.67
Topo II	30	21	3	2	4	20.00
GST-π	30	13	14	2	1	10.00
MRP	30	24	4	1	1	6.67
LRP	30	22	6	1	1	6.67

The expression of resistance proteins in interstitial cells are similar to the tumor cells. The positive expression of P-gp is highest, the difference was statistically significant (Rank sum test, P < 0.05)

### Expression of the 5 multidrug resistance proteins in different grade tumors

In tumor cells and interstitial cells, there was no significant difference between the expression of the 5 multidrug resistance proteins (Fisher definite probability methods, P > 0.05) between high grade and low grade tumors (Tab 4). In the capillary vessels, the strong positive expression of P-gp was 60% (6/10) in high grade and 10% (2/20) in low grade tumors. This difference was statistically significant (Fisher definite probability methods, P < 0.05) (Tab 4), which shows that the P-gp positive rate in high grade tumors is higher than in low grade tumors and in capillary vessels.

**Table 4:** Positive expression of the 5 multidrug resistance proteins in the different grades of brain tumors

Multidrug resistance proteins	Tumor cells			Capillary vessels			Interstitial cells		
			
	H	L	P	H	L	P	H	L	P
n	10	20	-	10	20	-	10	20	-
P-gp	4	3	0.378	6	2	0.027	8	19	0.251
Popo	4	4	0.384	0	0	-	0	0	-
GST	3	2	0.3	0	0	-	8	14	0.682
MRP	0	0	-	0	0	-	2	2	0.584
LRP	0	0	-	0	0	-	2	2	0.584

In tumor cells and interstitial cells, there was no significant difference between the expression of the 5 multidrug resistance proteins (Fisher definite probability methods, P > 0.05) between high grade and low grade tumors. In the capillary vessels, the strong positive expression of P-gp was 60% (6/10) in high grade and 10% (2/20) in low grade tumors. This difference was statistically significant (Fisher definite probability methods, P < 0.05).

### Double P-gp/caveolin-1 immunolabel

**Figure 2:**
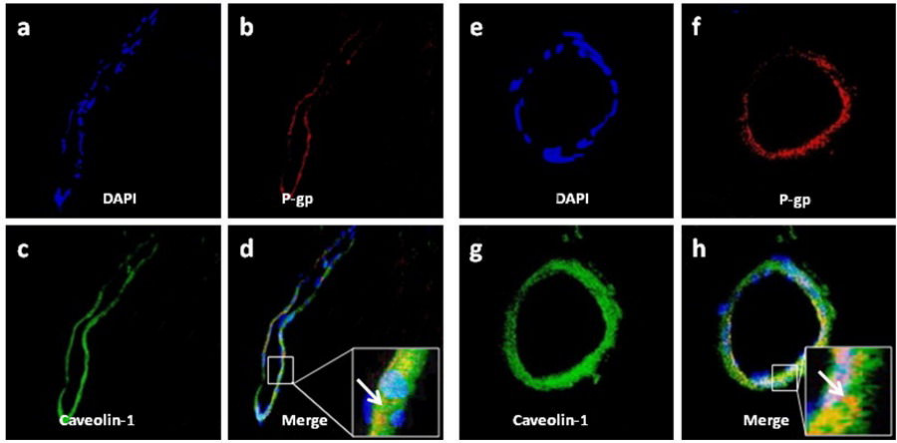
**Immune co-labeling of P-gp/caveolin-1 in capillary endothelial cells. (×40 ×2 zoom)**. (a, e) Nuclear staining. (b, f) P-gp labeling appears concentrated in the luminal compartment of the endothelial cells. (c, g) Caveolin-1 stains the entire endothelial cytoplasm with fine puncta in the luminal compartment and larger, intensely immunoreactive puncta in the abluminal compartment. (d, h) The merged images show P-gp and caveolin-1 co-expression (yellowish fluorescence). the two proteins co-localize either in the luminal endothelial compartment (d, arrow) or at the border of the luminal/abluminal compartments (h, arrow).

On the double P-gp/caveolin-1-immunolabeled samples, observation of sections at higher magnification on serial optical planes of cross-sectioned microvessels confirmed that the expression of P-gp corresponded to the endothelial cells and also revealed that the transporter is localized in the luminal compartment of endothelial cells (Fig 2b and Fig 2f). Unlike P-gp, caveolin-1 stained the entire thickness of the endothelium from the luminal to the abluminal side with a finely punctate pattern in the endothelial luminal compartment and larger fluorescent puncta in the abluminal luminal compartment (Fig 2c and Fig 2g). Comparing the single channel signal of P-gp and caveolin-1 (green and red fluorescence, respectively), it is evident that both proteins are present in the luminal compartment of the endothelial cells. Nevertheless, the exact extent of P-gp/caveolin-1 co-localization is only revealed on the merged images, which were obtained by superimposing the two fluorescent signals (Fig 2d and Fig 2h, yellow fluorescence). P-gp and caveolin-1 most frequently co-localized in the luminal compartment of the endothelial cells, although elsewhere, the fluorescent signals do not appear to overlap completely, and co-localization was detectable only at the boundary between the luminal and abluminal endothelial cell compartments.

## Discussion

A large number of studies have analyzed P-gp substrates, expression and activities in brain tumors. Cultures of cerebral endothelial cells, isolated brain microvessels, and the P-gp knockout mouse have been used to study the functions of P-gp. In the specific field of the human BBB, our study contributes to the knowledge of cellular localization and molecular interactions of P-gp in brain tumor tissue in situ. The results shown here indicate that P-gp is mainly expressed in the endothelial cells lining and surrounding small vessels, in which the transporter appears concentrated within the luminal cellular compartment. LRP, MRP, GST-π and Topo II are not expressed in the capillary vessels and are partly expressed in the interstitium. In order to identify the exact location of P-gp in the capillary vessels, immunostaining for S-100 protein was simultaneously performed. S-100 is expressed in glial and Schwann cells but is not expressed in capillary endothelial cells and basement membrane. Our results confirm that P-gp is located in the end-feet of glial cells. There were two pieces of evidence to support this. One, S-100 was observed in capillary vessels, and the localization of S-100 was similar to that of P-gp. Two, the localization of S-100 was consistent with P-gp localization in the interstitial tissue. In the intracranial region, most of the glial cells are astrocytes, and P-gp is located in the end-feet of the astrocytes. These data confirm an effective role of endothelial P-gp as a "gatekeeper" in the BBB that limits the influx of drugs in the brain and indicate the pericytes as a possible second line of defense at BBB sites[13]. So, whether before the drugs through the BBB or during the drugs through the BBB, P-gp has played an important role in multidrug resistant. In this study, we found that there was no difference in the expression of multidrug resistance proteins between different degrees of malignancy of brain tumor cells. However, there were significant differences in expression of these proteins in the capillary vessels, which suggests that the expression of multidrug resistance proteins in the capillary vessels is potentially the main reason for differential resistance in brain tumors with differing malignancies.

Our study also demonstrated that the expression of P-gp in the interstitial cells was related to the distance of the cells from the capillary wall. The nearer the cell was to the capillary wall, the stronger the expression of P-gp. That is, where there were a large number of tumor cells but no capillaries, no expression of P-gp in tumor cells and the interstitium was observed, which shows that the multidrug resistance of brain tumors mainly occurs in and around the capillaries and is related to the distance from capillaries.

Currently, part of the research on P-gp is focused on its localization in caveolae [14]. Caveolae are flask-shaped, invaginated membranes enriched in cholesterol and sphingomyelin, which confer particular physicochemical properties including insolubility in anionic detergents and low-buoyant density in sucrose gradients [15-17]. These microdomains are present in a wide variety of cell types and are dynamic structures involved in transcytosis, potocytosis and signal transduction [18]. Caveolin-1, one of the major structural protein of caveolae, co-localizes with P-gp in fractions of rat brain capillaries [11]. The expression of both P-gp and caveolin-1 is increased when cellular plasma membrane caveolae are increased [19,20]. Furthermore, by immunoprecipitation and immunofluorescence laser scanning confocal microscopy experiments, caveolin-1 has been demonstrated to physically interact with P-gp in the microvascular endothelium and at the extensive networks of astrocytic processes [11,21]. However, in brain tumors, there are few reports about the interaction between P-gp and caveolin-1.

The data reported in this study on the co-localization of P-gp with caveolin-1 provide the morphological evidence of the association between P-gp and caveolin-1 in brain tumor endothelia and highlight the dynamic nature of this interaction. For the studies on caveolin-1 and P-gp distribution and colocalization, major points have to be considered. The studies use immunolabeling of brain tissues with antibodies against P-gp and caveolin-1, and evidence was found for the expression of P-gp on the luminal membrane of the capillary endothelium in brain tumors. However, caveolin-1 is expressed on the entire thickness of the endothelium from the luminal to the abluminal side. P-gp and caveolin-1 appear to largely co-localize in the luminal compartment of the endothelial cells, and co-localization is detectable only at the boundary between the luminal and abluminal endothelial cell compartments. A previous study demonstrated that only a portion of P-gp molecules [11] are associated with caveolin-1, which suggests that different cell phenotypes may modify the localization of P-gp and caveolin-1, and different cellular events may lead to redistribution of both proteins.

In summary, the present study indicates that P-gp is mainly expressed in capillary endothelial cells and end-feet of glial cells. P-gp, an important part of the blood brain barrier, plays a significant role in brain tumor resistance. In addition, the expression of P-gp in the interstitial cells was related to the distance of the cells from the capillary wall. The nearer the cell was to the capillary wall, the stronger the expression of P-gp. In the brain, the expression of P-gp and caveolin-1 was found at both the end-feet of astrocytes and microvascular endothelium. The parallel expression of P-gp and caveolin-1 supports the hypothesis that these two transporter proteins may work in concert to mediate transport processes in the brain at several levels, including the microvascular endothelium, the microvascular astrocytic end-feet, and parenchymal astrocytic processes.

## Competing interests

The authors declare that they have no competing interests.

## Authors' contributions

ZG collected the clinical datas and samples, participated in the immunohistochemistry and drafted the manuscript. JZ carried out the immunohistochemistry. LZ performed the statistical analysis. QL participated in the design of the study. XJ conceived of the study, and participated in its design and coordination. All authors read and approved the final manuscript.
